# Patients’ and clinicians’ expectations on integrative medicine Services for Diabetes: a focus group study

**DOI:** 10.1186/s12906-020-02994-5

**Published:** 2020-07-02

**Authors:** Kam Wa Chan, Pak Wing Lee, Crystal Pui Sha Leung, Gary Chi Wang Chan, Wai Han Yiu, Hoi Man Cheung, Bin Li, Sarah Wing Yan Lok, Hongyu Li, Rui Xue, Loretta Yuk Yee Chan, Joseph Chi Kam Leung, Tai Pong Lam, Kar Neng Lai, Sydney Chi Wai Tang

**Affiliations:** 1 Division of Nephrology, Department of Medicine, The University of Hong Kong, Hong Kong, Hong Kong SAR; 2 Department of Family Medicine and Primary Healthcare, Hong Kong East Cluster, Hospital Authority, Hong Kong, Hong Kong SAR; 3 Division of Nephrology, Department of Medicine, Queen Mary Hospital, Hong Kong, Hong Kong SAR; 4 Department of Family Medicine and Primary Care, The University of Hong Kong, Hong Kong, Hong Kong SAR

**Keywords:** Qualitative, Expectation, General practice, Internal medicine, Integrative medicine, Diabetes

## Abstract

**Background:**

Difference of perspective between patients and physicians over integrative medicine (IM) research and service provision remains unclear despite significant use worldwide. We observed an exceptionally low utilisation of IM and potential underreporting in diabetes. We aimed to explore the barriers and recommendations regarding service delivery and research of IM service among diabetes patients and physicians.

**Methods:**

A 10-group, 50-participant semi-structured focus group interview series was conducted. Twenty-one patients with diverse severity of disease, comorbidities and education levels; and 29 physicians (14 conventional medicine (ConM) and 15 Chinese medicine (CM)) with diverse clinical experience, academic background and affiliation were purposively sampled from private and public clinics. Their perspectives were qualitatively analysed by constant comparative method.

**Results:**

Seven subthemes regarding barriers towards IM service were identified including finance, service access, advice from medical professionals, uncertainty of service quality, uncertainty of CM effect, difficulty in understanding CM epistemology and access to medical records. Patients underreported the use of CM due to the concern over neutrality of medical advice among physicians. Inconvenience of service access, frequent follow-up, use of decoction and long-term financial burden were identified as key obstacles among patients. Regarding research design, ConM physicians emphasised standardisation and reproducibility while CM physicians emphasised personalisation. Some CM-related outcome measurements were suggested as non-communicable. Both physicians acknowledged the discordance in epistemology should be addressed by pragmatic approach.

**Conclusion:**

Key obstacles of CAM clinical utilisation are different between patients. Further assessment on IM should be pragmatic to balance between standardisation, reproducibility and real-world practice. Evidence-based IM programs and research should merge with existing infrastructure.

## Background

Controversies on incorporating complementary and alternative medicine (CAM) into integrative medical (IM) care continue amid increasing utilisation and volume of evidence worldwide [1–5]. It was estimated that 25.9% of the general population in Europe in 2014 [6] and 35% of US adult population used CAM in 2015 [7], associated with higher education and income [6, 7]. The main form of CAM used were body-mind therapies and herbal treatments, and Chinese medicine (CM) is one of the most utilised streams of CAM [6, 7]. Patients with chronic ‘incurable’ conditions tend to perceive CAM as an efficacious alternative [8, 9].

Hong Kong has a developed health system and the introduction of CM faces challenges similar to the western world [10, 11]. While the lack of research-related capacity (mainly quality of evidence) and cultural believe has been repeatedly identified as key obstacles in existing literature [12–14], barriers and recommendations beyond these two domains requires further studies, especially from the implementation and organisational perspective [8, 15]. Also, research on the comparison of the perspectives among patients and physicians, especially between physicians from CAM and conventional medicine (ConM) of different specialties is limited amid the global call on addressing misaligned expectations between stakeholders prior to the design of interventions [16–19].

Diabetes is an alarming pandemic affecting 9.5% world adult population, accounting for 9.9% of global all-cause mortality [20, 21]. Diabetes is estimated to cost US$ 850 billion worldwide in 2017, accounting for 11.6% of global health expenditure [20, 21]. Diabetic kidney disease (DKD) refers to the chronic kidney disease (CKD) attributable to diabetes. DKD develops among 25–40% of all diabetic patients [22–24] and is presented in 2–3% of general population worldwide [25–27]. DKD is the major contributor to morbidity and mortality among diabetic patients which awaits more treatment options [22–24, 28–30]. A series of recent big data studies have shown the renal benefit of CM in reducing risk of end-stage kidney disease and mortality [31–33]. Different CM formulations have been reported to protect against diabetes and DKD via orchestrated mechanisms [5, 31, 32, 34–36].

Nevertheless, a service programme in Hong Kong revealed that only 20% of diabetes patients have ever considered CM as an option and only less than 2% have ever used CM as a treatment for diabetes or DKD which was way below the utilisation in other conditions (e.g. around 50% for cancer patients) [37]. Interestingly, some patients reported that ConM and CM physicians both discouraged IM care. The aristolochic acid-related herbal nephrotoxicity [38, 39] and over-the-counter CM-associated increase all-cause mortality [40] may partly explain the reluctance of ConM – CM collaboration in DKD [12]. However, the exceptional underutilisation and potential underreporting of CAM use [41] in diabetes hinted at possible unidentified expectation mismatch between CM, ConM physicians and patients from existing literature that is specific to the condition and worthy of special attention.

We aimed to explore the barriers and recommendations regarding IM service delivery among patients and physicians of conventional medicine and CAM, and subsequently identify key areas for research, education and service provision.

## Methods

### Study design

We conducted a 10-group semi-structured focus group interview series on patients and physicians with constant comparative method. Participants were recruited from public and private clinics and teaching hospitals in Hong Kong.

### Participants

Sixty-one invitations were sent out. One patient rejected due to family issues and another 2 rejected due to difficulties in allocating time for the study. Five CM physicians rejected due to work engagement and 3 did not respond to email invitations.

Fifty subjects were recruited (21 patients, 14 ConM physicians and 15 CM physicians) from October 2015 to June 2018. Patients aged 18 or above, diagnosed with diabetes / DKD and having follow-up at public outpatient clinics were included and sampled purposively from the consultations of general, renal and family medicine outpatient clinics of Queen Mary Hospital and Tang Shiu Kin Hospital to form 3 groups of 6–8 with diverse age groups, CKD stages, diabetes duration, co-morbidities and education levels to enrich data coverage.

ConM physicians formed 3 groups of 3–6, and CM physicians formed 4 groups of 3–4. Physicians with experience in managing diabetes / DKD and were included and purposively sampled with diverse age groups, practicing duration, qualification and experience of ConM and CM, affiliating institutions, service sector (private / public) and involvement in research, administration and teaching.

Participants were recruited face-to-face, though telephone or by email. The purpose of interview was explained to the participants by the research coordinator (K.W.C.). The process of recruitment, interview and analysis were iterative until data saturation was observed.

### Interview process and interview guide

The focus group interviews lasted 60–120 min (allowing at least 20 min time per participant per group for sufficient participation) and were conducted privately in meeting places near participants’ workplace or residence.

The interviews were facilitated by a moderator (P.W.L.) with 1) undergraduate public health training, 2) 2 years of in-depth interviews and focus group interviews moderation experience and 3) basic knowledge on CM, ConM and diabetes to ensure adequate skills and knowledge for conducting the interviews. The moderator was not involved in the conceptual design of the study, did not have prior contact with the study subjects, did not have conflict of interests with subjects and the research topic, and was informed about the group composition until 1 week before the interview. The identity of moderator was not disclosed to participants before interviews. He was working in public health administration sector but not involved in integrative medicine or diabetes issues in daily work during the period of study to avoid any potential conflict of interests. One to two clerical staffs were present at different interview sites for administrative support. The moderator recorded participants’ demographics with simple questionnaire and mediated the interviews.

The discussion was built around consultation experience, concerns and expectations based on a semi-structured interview guide (Supplementary File). The interview guide was piloted among other patients and physicians prior to the interviews. Minor ad-hoc adjustment was made to explore new themes. Patient group started prior to physician groups in each round of interview. In the first and early second round of interviews, the interviews focused on gathering detail experience in consultation encounters. As comments began to repeat in second round, emphases were put on contrasting previous groups’ findings across subject groups (e.g. discussing findings of patient groups with physicians) and on seeking solutions. Data saturation was observed during the last round (patient and ConM: 3rd round, CM: 4th round) of interview in the respective groups and therefore sampling ceased [42, 43].

Interviews were audiotaped and transcribed verbatim. Interviewees’ identity was preserved by using individual codes. Brief notes on interview content and behaviour of interviewees were made real-time during interviews and subsequently combined with the transcripts. The transcripts were cross-checked between 2 independent researchers (K.W.C., P.W.L.).

### Data collection and analysis

The transcripts were analysed using the constant comparative method [44] aided by simple software (Microsoft Word and Excel) for better access. The transcripts were first familiarised independently by 2 bilingual investigators (K.W.C., P.W.L.). Maximum codes on main themes and subthemes were generated independently to open up all fields during initial open coding. The transcripts were revisited to check for emerging ideas. The concepts and theories were refined, and the association of the coding was explored to form axial coding. A preliminary coding scheme was established with agreement by investigators. The preliminary findings were discussed with one representative from each group for validation and supplementation based on the interview content [45].

**Fig. 1:**
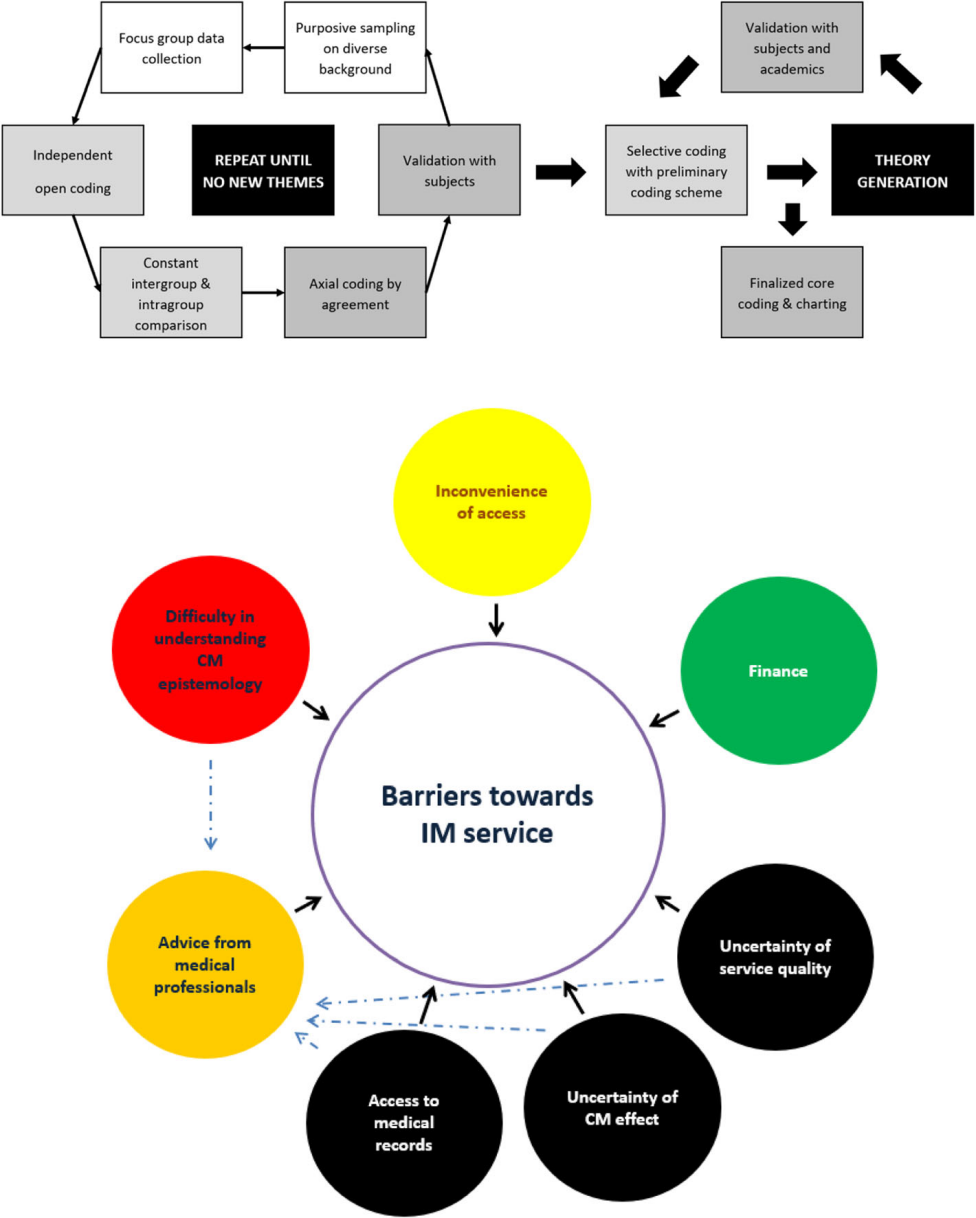
Coding process and the relationship between subthemes and barriers towards integrative medical service

Core coding was confirmed for the dataset after observing no new themes at the last round of interview. The finalised coding scheme was applied to index and chart the whole dataset. Charted result was translated by a bilingual investigator (K.W.C.) when used as illustrative quotations to best preserve original meaning. Analysis was completed in October 2018. The analysis process is summarised in Fig. 1.

## Results

### Demographics

Demographics of subjects are presented in Tables 1 and 2. Majority of patients had unsatisfactory glycemic control (71.4%), CKD stage 2–4 (95.2%) and albuminuria (90.5%). Eight (57.1%) ConM physicians were internists and 6 (42.9%) were family medicine specialists or general practitioners. 6 (42.9%) ConM physicians had informal CM education and all CM physicians (*n* = 15) had formal credit-bearing ConM education in anatomy, physiology, immunology, pathology and internal medicine as CM undergraduate programmes contained substantial portion of ConM elements in Hong Kong. Table 3 presented the CM and ConM education background of participants.

**Table 1:** Baseline demographics of patients

Characteristics	Patient (***n*** = 21)
**Age group – no. (%)**	
**41 to 60**	3 (14.3)
**61 or above**	18 (85.7)
**Male ratio – no. (%)**	13 (61.9)
**Education level – no. (%)**	
**No formal education**	1 (4.8)
**Primary education**	8 (38.1)
**Secondary education**	8 (38.1)
**Tertiary education**	4 (19.0)
**Employment status – no. (%)**	
**Part-time employed**	1 (4.8)
**Full-time employed**	7 (33.3)
**Unemployed or retired**	13 (61.9)
**History of diabetes – yrs**	12 ± 5.6
**Hemoglobin A1C – %**	7.2 ± 0.7
**Unsatisfactory control (7% or above) – no. (%)**	15 (71.4)
**Chronic kidney disease stage – no. (%)**	
**Stage 2 (60 ml/min/1.73m**^**2**^**or above)**	7 (33.3)
**Stage 3a (45 to 59 ml/min/1.73m**^**2**^**)**	5 (23.8)
**Stage 3b (30 to 44 ml/min/1.73m**^**2**^**)**	3 (14.3)
**Stage 4 (15 to 29 ml/min/1.73m**^**2**^**)**	5 (23.8)
**Stage 5 (below 15 ml/min/1.73m**^**2**^**)**	1 (4.8)
**Urine albumin-to-creatinine ratio – no. (%)**	
**< 3.4 mg/mmol (normo-albuminuria)**	2 (9.5)
**3.4 to 34 mg/mmol (micro-albuminuria)**	10 (47.6)
**> 34 mg/mmol (macro-albuminuria)**	9 (42.9)
**Comorbidity – no. (%)**	
**Hypertension**	21 (100)
**Dyslipidemia**	19 (90.5)
**Diabetic retinopathy**	6 (28.6)
**Coronary artery disease**	1 (4.8)
**Major form of conventional medicine care received – no. (%)**	
**Primary care**	15 (71.4)
**Secondary care**	6 (28.6)
**Chinese medicine service experience – no. (%)**	
**Attended Chinese medicine consultation before**	14 (66.7)
**Encountered suspected Chinese medicine-related untoward events**	2 (9.5)
**Awareness of chronic kidney disease status – no. (%)**	13 (61.9)

In mean ± SD. Patients with end-stage kidney disease, macroalbuminuria, other co-morbidities and encountered suspected Chinese medicine-related untoward events were purposively sampled for the third-round interview

**Table 2:** Baseline demographics of physicians

	Conventional medicine (ConM) physician (***n*** = 14)	Chinese medicine (CM) physician (***n*** = 15)
**Age group – no. (%)**		
**Below 30**	3 (21.4)	2 (13.3)
**31 to 40**	8 (57.1)	12 (80)
**41 to 50**	2 (14.3)	1 (6.7)
**51 to 60**	1 (7.1)	0 (0)
**Male ratio – no. (%)**	9 (64.3)	11 (73.3)
**Full-time employed – no. (%)**	14 (100)	15 (100)
**Years of post-qualification practice – no. (%)**		
**5 years or below**	2 (14.3)	1 (6.7)
**6 to 10 years**	1 (7.1)	8 (53.3)
**11 to 15 years**	7 (50)	6 (40)
**16 to 20 years**	2 (14.3)	^e^ 0 (0)
**20 years or more**	2 (14.3)	^e^ 0 (0)
**ConM – Specialist qualification – no. (%)**	9 (64.3)	^f^ N/A
**CM – Postgraduate doctoral clinical training – no. (%)**	N/A	7 (46.7)
**CM – Postgraduate doctoral training place – no. (%)**		
**Hong Kong**		3 (20)
**Mainland**		3 (20)
**Concentration of practice – no. (%)**		
^**a**^**Family medicine**	6 (42.9)	15 (100)
^**b**^**Internal medicine**	8 (57.1)	^f^ N/A
**Service provision – no. (%)**		
**Primary care**	6 (42.9)	15 (100)
**Secondary care**	8 (57.1)	^f^ N/A
**Affiliation**		
**Public health clinical care system**	13 (92.9)	^f^ N/A
**Non-governmental organisation**	0 (0)	5 (33.3)
**University**	1 (7.1)	7 (46.7)
**Private practice**	0 (0)	3 (20)
^**c**^**Actively engaged in medical research**	5 (35.7)	8 (53.3)
^**d**^**Actively engaged in medical management**	4 (28.6)	11 (73.3)

^a^ including general practice

^b^ including endocrinology, hepatology and nephrology

^c^ including basic science, clinical, policy and other types of medical research

^d^ holding appointments other than clinical position

^e^ Chinese medicine (CM) undergraduate education and registration started in Hong Kong in 1997

^f^ there is no specialist registration system for CM physicians and no public CM service in Hong Kong. Conventional medicine physicians with over 20 years practicing experience, affiliated to university, and managed suspected CM untoward events were purposively sampled for second and third round

**Table 3:** Background of Chinese medicine and conventional medicine education among study subjects

Chinese medicine (CM) / Conventional medicine (ConM) background – no. (%)	Patient (***n*** = 21)	ConM physician (***n*** = 14)	CM physician (***n*** = 15)
^**g, i**^**Formal CM education**	0 (0)	1 (7.1)	15 (100)
^**g, j**^**Informal CM education**	3 (14.3)	6 (42.9)	
^**h, i**^**Formal ConM education**	0 (0)	14 (100)	15 (100)
^**h, j**^**Informal ConM education**	5 (23.8)		15 (100)

^g^ attended any Chinese medicine (CM) theory-related sessions including Chinese herbal medicine, acupuncture, moxibustion, orthopedics and health management

^h^ attended any biomedical or epidemiological sessions involving clinical medicine

^i^ defined as credit bearing sessions

^j^ defined as non-credit bearing sessions including health talks, seminars and other forms of information sessions. All CM physicians were exposed to formal conventional medicine (ConM) education included but not limited to anatomy, physiology, microbiology, immunology, pathology, diagnosis, pharmacology and internal medicine. Postgraduate doctoral clinical training was presented as a comparable qualification of specialist training of CM physicians as there is no CM specialist registration system in Hong Kong. Patients with informal CM and ConM educations were purposively sampled for second and third round. ConM physician with formal CM education were purposively sampled for second round

### High-level themes

Seven high-level themes (barriers towards IM service, motivation to seek CM service, background knowledge on diabetes, experience of CM service, preferred model of integrative service delivery, evidence of IM and CM hospital) were identified with 25 subthemes. Data on barriers towards IM service and preferred model of integrative service delivery are summarised in Fig. 1. Additional quotes are summarised in Table 4.

**Table 4:**
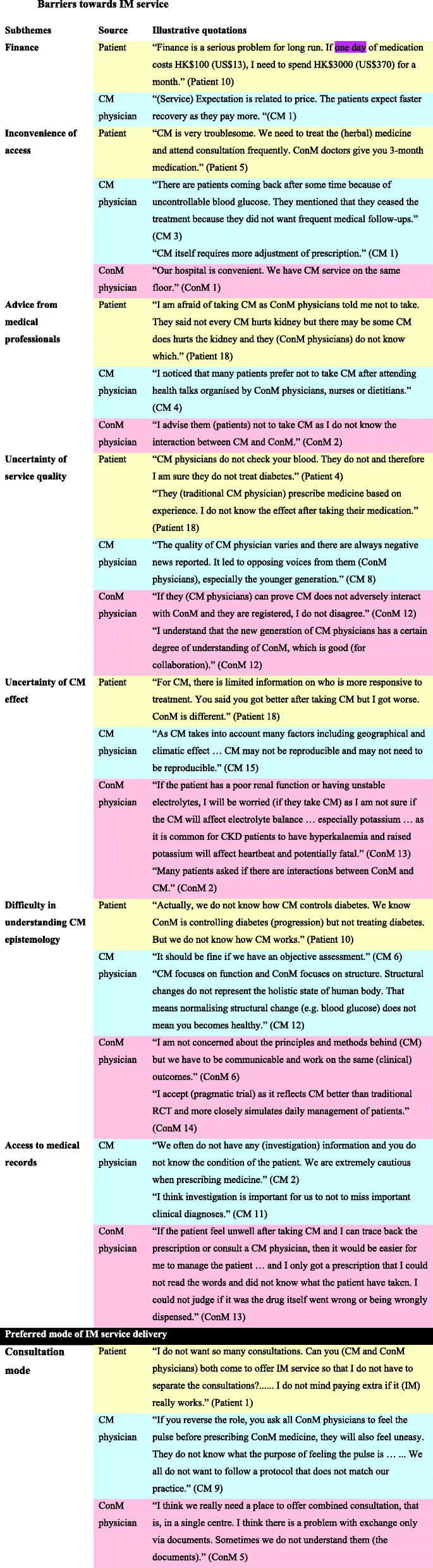
Subthemes and illustrative quotations of the main theme

Perspectives of patients, conventional medicine (ConM) physicians and Chinese medicine (CM) physicians were compared

Themes generally agreed by patients in yellow, by Chinese medicine (CM) physicians in blue, by conventional medicine (ConM) physicians in red, by both patients and CM physicians in green, by both patients and WM physicians in orange, by all parties in black

#### Theme 1: barriers towards IM service

Seven subthemes were identified regarding barriers towards IM service, including finance, inconvenience of access, advice from medical professionals, uncertainty of service quality, uncertainty of CM effect, difficulty in understanding CM epistemology and access to medical records.

##### Finance

Finance was identified as a major practical issue against the use of CM / IM by patients. CM service in Hong Kong is financed through insurance and out-of-pocket payment. Government subsidised over 90% for ConM service [46] and comparable CM service for diabetes / DKD operated by non-governmental organisations is around 100 times more expensive [47]. The chronic nature of diabetes / DKD intensified the impact of finance.

##### Inconvenience of access

The inconvenience of access to CM service, including frequent consultation and the use of raw herbs was also frequently mentioned among patients, although majority of them (61.9%) were unemployed / retired. Patients had a longer follow-up period of 3–6 months for ConM services when compared to 1–2 weeks for CM. Although CM physicians anticipated that inconvenience would affect compliance, they believed it was inevitable due to the personalised nature of CM.

*“CM is very troublesome. We need to treat the (herbal) medicine and attend consultation frequently. ConM doctors give you 3-month medication.” (Patient 5)*

##### Advice from medical professionals

While some patients were advised against CM by ConM physicians, they hesitated to comply as they believed ConM physicians could not decide the effect of CM. We sought to delineate the underlying mechanism of discouragement and the lack of understanding of CM effect (especially on the interactions of IM) emerged as the key reason and is further dissected.

*“They (ConM physicians) do not know which CM physician you consulted and do not know what CM you have taken. How can they give an answer?” (Patient 10)*

*“I advise them (patients) not to take CM as I do not know the interaction between CM and ConM.” (ConM 2)*

##### Uncertainty of service quality

Uncertainty of service quality was a common concern among patients, ConM and CM physicians. Patients doubted CM physicians’ academic qualification and questioned the experience-based consultation of some CM physicians.

*“They (traditional CM physician) prescribe medicine based on experience. I do not know the effect after taking their medication.” (Patient 18)*

ConM physicians were concerned about the standardisation of CM, interaction between CM and ConM and the background nephrology knowledge among CM physicians.

*“If they (CM physicians) can prove CM does not adversely interact with ConM and they are registered, I do not disagree.” (ConM 12)*

*“I advised them (patients) not to approach CM physicians who are not affiliated with hospitals or NGOs as I am not sure about their understanding on kidney disease or replacement therapy.” (ConM 14)*

##### Uncertainty of CM effect

All parties were uncertain about the efficacy of CM. A patient who experienced suspected CM nephrotoxicity suggested that the response to CM was personalised, as he experienced toxicity despite others’ positive feedback. CM physicians also acknowledged that there may not be adequate evidence to inform specific management. However, there were ambivalent opinions on the study design for further research, especially on clinical trials.

*“For CM, there is limited information on who is more responsive to treatment. You said you (another patient participant) got better after taking CM but I got worse. ConM is different.” (Patient 18)*

*“As CM takes into account many factors including geographical and climatic effect … CM may not be reproducible and may not need to be reproducible.” (CM 15)*

##### Difficulty in understanding CM epistemology

ConM physicians well-noted that CM has different theory basis and clinical practice when compared to ConM. All parties agreed to emphasise more on the clinical effectiveness.

*“I studied a short course of CM … the fundamental concept of CM and ConM is very different. For example, when CM refers to heart, lung, liver, spleen and kidney, it is not the organ we understand, they link to five elements and ying yang which is hard to directly translate to ConM.” (ConM 3)*

*“I am not concerned about the principles and methods behind (CM) but we have to be communicable and work on the same (clinical) outcomes.” (ConM 6)*

However, ConM physicians suggested that it is hard to understand and translate CM-related clinical outcomes to their practice.

*“(CM theory) is difficult to me. Even if they (CM physicians) wrote consultation notes, I could not understand … I do not understand how they monitor disease progress.” (ConM 1)*

##### Access to medical records

ConM physicians raised concern that the CM medical record, especially hand-written prescription, was often unreadable. This imposed a risk of mishandling of patients under CM service. Also, ConM physicians were worried that the restricted access of investigations among CM physicians may affect clinical management. However, CM physicians who advocated traditional practice were less relied on investigation while other CM physicians suggested that investigations would help alert them critical conditions that require prompt management.

*“I am worried that CM does not have investigations.” (ConM 14)*

#### Theme 2: preferred model of IM service delivery

Four subthemes including organisational management, consultation mode, CM-ConM communication and choice of CM service were identified under the theme ‘Preferred model of IM service delivery’. Data on consultation mode is presented below.

*“I do not want so many consultations. Can you (CM and ConM physicians) both come to offer IM service so that I do not have to separate the consultations?...... I do not mind paying extra if it (IM) really works.” (Patient 1)*

##### Consultation mode

Majority of participants suggested that there was a need of physically combined service, preferably at the same institution. This would improve the confidence of patients and communication between physicians, leading to more efficient patient management. Patient also expected physicians to offer compromised IM treatment protocol as clinical solutions. Both ConM and CM physicians acknowledged that the combined treatment should be formulated according to the appropriate theory basis, i.e., CM treatment directed by CM theory and *vice versa*.

*“If you reverse the role, you ask all ConM physicians to feel the pulse before prescribing ConM medicine, they will also feel uneasy. … ... We all do not want to follow a protocol that does not match our practice.” (CM 9)*

*“I think collaborative service has a great potential but theory integration almost looks impossible. Even if we have a pharmacy offering both ConM and CM, it does not mean that CM physicians would accept us (ConM physicians) to prescribe CM based on ConM theory. Vice versa, it would be hard for us to accept CM physicians prescribing mycophenolic acid because the patient is having ‘qi deficiency’.” (ConM 7)*

## Discussion

This is the first focus group study comparing the expectation of patients and physicians regarding the use of IM service for diabetes, with the participation of family medicine, internal medicine and CAM physicians in addition to patients. We demonstrated that there was an expectation mismatch between patients (prefer physicians to design integrative treatment protocol and prefer biomarkers for disease monitoring), CAM physicians (believed diabetes patients focused on quality of life) and physicians of conventional medicine (prefer patients to make the choice of treatment).

### Justification of study design

We investigated with a qualitative approach to explore unknown mechanism in the underutilisation and underreporting of CAM among diabetes patients and physicians and conducted focus group interviews to enhance interactions between participants with different experience. The interview guide was designed to explore more practical concerns and actual consultation experience from implementation perspective as existing literature is limited in this aspect. The guide was slightly tilted towards exploring how to integrate CM service into existing ConM system as majority of diabetes patients were receiving ConM as background treatment locally and internationally. The bias of interviewing skills and contents was minimised with an independent experienced moderator. Patients and physicians with different background hypothesised to offer divergent perspectives (e.g. complications for patients, training and work nature for physicians) were purposively sampled to enrich the content and coverage. As possible underreporting of IM utilisation was previously reported [41], we separated patients and physicians into different groups to encourage expression of comments and experience, and to minimise dominant opinion.

Data saturation, defined by having no emerged new key themes, was reached in the third round of interview among patients and ConM physicians, and the fourth round of CM physician interview. This reconciled with previous review and empirical studies that 80% of themes could be captured in 2 to 3 rounds of interview and data saturation usually occurred in the 3 to 4 rounds [42, 43]. Only two high level themes were presented as this was a large-scale focus group study and we are presenting the most relevant themes and subthemes to maintain focus in discussion.

### The unaddressed and less documented preference and practical concerns

Existing literature regarding the obstacles of IM emphasised cultural believe and research-related capacity. Our study echoed as uncertainty of service quality and effect of CM were identified as shared concerns among all stakeholders as summarised in Fig. 1.

Our findings further highlighted the importance of mutual understanding between physicians of different streams as any advices regarded unreasonable and unexplainable by patients would result in non-compliance and underreporting. In addition to the existing understanding, practical issues including inconvenience of access, frequent follow-up and financial burden were stressed among patients in this series, which were less documented and is likely related to the chronic nature of diabetes. Besides, we found that ConM with exposure to CM knowledge preferred more pragmatic and clinical evaluation of IM service as they recognise there is a fundamental difference in epistemology between ConM and CM. We also documented more practical concerns from the medical community for the consideration of further IM study designs.

### Implications for practice, organisation and policy

Convenience of access was frequently quoted by patients as a key barrier while it was not concerned by CM physicians. IM would lead to increased number of clinical visits. While frequent follow-up may provide patients with the best-tailored management, the required effort would compromise patients’ quality of life and increase the economic loss. Combined IM consultation under the same organisational structure was generally preferred by our subjects (both patient and physicians) as it enhances quality control, reduces time cost for consultation and facilitates sharing of medical records among physicians. Integrated clinics are increasingly common globally and provide a platform for physicians from different streams to design and evaluate IM protocols [48]. High quality decocting-free herbal granules provide patients more standardised and user-friendly administration for long-term use [49].

In contrast to many existing IM models globally that the decision and liability of IM use was shifted to the patients, requiring patients to actively search and critique the evidence themselves, our data suggested that patients expected physicians of different streams to optimise the IM protocol and offer integrated evidence-based recommendations for their informed decision making. This will require centralised and concerted effort in evaluating IM efficacy with parallel mechanism of existing statutory bodies, for instance NICE (UK), FDA (US) and NMPA (China), if not integrating IM procedures into these existing infrastructures.

Interestingly, CM physicians from private sector believed the access cost of CM has driven patients to demand extra efficacy and quantifiable evidence when evaluating CM, which amplified the effect of lack of evidence, a well-known barrier. Patients reconciled that cost was one of the key barriers of using IM service. In Hong Kong, CM service is mainly financed through out-of-pocket payment and private insurance whereas ConM service is over 90% publicly subsidised. Comparable CM service of diabetes operated by NGOs is around 100 times more expensive in long run. Government policy on CAM service financing emerged as a decisive factor of implementation.

### Implications for education and research

Physicians from both sides called for more basic IM knowledge from their counterparts for better communication. In China, most physicians are double qualified and practise ConM and CM simultaneously. While dual training may provide a comprehensive foundation for IM practice, there are criticisms on the ungrounded integration between ConM and CM on physician level as suggested by the interviewees. As many ConM and CM interventions have been shown to have interactions, IM service requires specifically designed IM-oriented research and evidence instead of simple integration of disaggregated evidence from either side. Also, dual training is difficult to propagate to health systems of other countries and requires substantial resources. The effectiveness and optimal ratio of one physician – dual medical practice model and one administrative system – dual streams of physician model in health systems also require further implementation studies.

Regarding research, both ConM and CM physicians acknowledged that research design should formulate according to the corresponding school of theory of ConM and CM to reflect actual practice. Nevertheless, ConM physicians emphasised more on standardisation and reproducibility while CM physicians focused more on personalisation on study design which highlighted the different epistemology between CM and ConM. Both CM and ConM physicians of different specialties proposed pragmatic trial as an agreeable solution which aligned with the trend of establishing real-world comparisons lately [19, 43, 50]. Patient selection, intervention, outcome measurement and analysis of the IM studies should take into consideration the real-world practice of different streams of medicine and patient preference.

### Limitations

This study has several limitations. Since the aim of this series was to identify detailed barriers, concerns and expectations on IM diabetes management, the findings are context specific, similar to other qualitative studies. However, we believe the major findings could be carefully generalised to the IM management of chronic conditions in places where CAM is not part of the public health system, including most of the developed countries. Also, this series only identified possible mechanisms of social behaviour and further quantitative studies including survey are needed to better determine the magnitude of and prioritisation of the concerns. Lastly, the study period was long due to the availability of funding and difficulties in organising focus groups, especially for physicians, which is a commonly known challenge.

## Conclusion

Inadequate explanation of CAM effect to patient may lead to underreporting. Practical issues on the access and finance of medical service emerged as major barriers to the delivery of IM service from the patients’ perspective. Inadequacy in evidence of clinical effectiveness and the difficulties in interpreting CM theory remained as the major obstacles in the consideration of CM among ConM physicians. Pragmatic clinical evaluation is mutually accepted by both ConM and CM physicians. Research design should focus on the add-on effect of CAM and consider standardisation, reproducibility and the real-world practice. To enhance the utilisation, IM programmes that are evidence-based should merge with existing infrastructure.

## Supplementary information

**Additional file 1.**

## Data Availability

The datasets used and/or analysed during the current study are available from the corresponding author on reasonable request.

## Abbreviations

CAM: Complementary and alternative medicine
CKD: Chronic kidney disease
CM: Chinese medicine
DKD: Diabetic kidney disease
IM: Integrative medicine / medical
ConM: Conventional medicine
