# Supplementary material for: Patients’ and clinicians’ expectations on integrative medicine Services for Diabetes: a focus group study
Source: BMC Complement Med Ther. 2020 Jul 2;20:205. doi: 10.1186/s12906-020-02994-5 (PMC7331247; doi:10.1186/s12906-020-02994-5)
Supplement: Supplementary file 1 — Additional file 1. [file 12906_2020_2994_MOESM1_ESM.pdf]

Supplementary File. Interview Guide

The perspective of patients and clinicians on diabetic kidney disease Chinese-western medicine referral

Interview Guide

A. Registration and preparation (5-10 mins):

1. Explain the interview purposes, confidentiality and the rights of participants (if needed)
2. Record name and HKID, assign subject ID (01, 02... etc)
3. Sign informed consent
4. Sign travel allowance reimbursement form

B. Introduction (5-10 mins):

1. Greeting and self-introduction
2. Participant round table introduction (Surname)
3. Recap of the interview purposes, confidentiality and the rights of participants
4. Ground rules for the interview
   a. No 'right' or 'wrong' answers, encourage diversity and no pressure
   b. More daily life examples
   c. Will be audiotaped
   d. 1 person speaking at each time
   e. Mobile devise switch to silent mode if possible

C. Interview (around 60 mins)

Abbreviation:

1. **CM = Chinese medicine**
2. **WM = Western medicine**
3. **IM = Integrative medicine**
4. **DM = Diabetes mellitus**
5. **DKD = Diabetic kidney disease**

Supplementary File

For WM physician:

1. Experience on managing DM / DKD patients and CM (ice-breaking, 5-10 mins)
   i. Is it frequent to encounter diabetes patients?
   ii. Is nephropathy common among them?
   iii. Are you interested in CM therapy?

2. Experience on referral of diabetes / diabetic kidney disease to CM physician (around 30 mins)
   i. Have you encountered any patients enquiring about the use of CM for DM or related complications?
      - Frequency (many?)
      - What do they ask? (Referral? Efficacy? Safety?)
      - Patient's stage of disease (with / without complications, CKD stage?)
      - Did they mention any particular herbs?
   ii. How would you feel about referral / use of CM and answer the patients in daily practice?
      - Do you feel comfortable for referral?
   iii. Have you encountered any patients with undesired medical behaviour (eg. Reluctant to comply with advice) / suspected CM induced complications?
      - How do they present?
      - How would you determine the relationship?

3. Concern on referral (around 30 mins)
   i. How do you perceive the effect and toxicity of CM / WM for diabetes and diabetic kidney disease in general?
   ii. What would you consider in advising patient referral for diabetes?
      - Any obstacles in consideration? Any further information / assistance / tools needed?
   iii. What do you expect the role / attitude of / contribution from CM physician?
   iv. Any suggested research area for CM / WM?
      - Common herbs?
      - Herbs that are likely to be / reported to be nephrotoxic?
      - Clinical protocol?
      - Responder analysis?
   v. Any suggested evidence dissemination form / platform?
      - Clinical practice guideline?
      - Web?
      - CME course / seminar?

4. Summary, conclusion and acknowledgement (5 mins)

Supplementary File

For CM physician (How to treat patients with diabetes from CM perspective is NOT the focus):

1. Experience on managing patients with diabetes / diabetic kidney disease (ice-breaking, 5 mins)
    i. Is it frequent to encounter patients with diabetes?
    ii. What are the common complications among them?

2. Experience on referral of patients with diabetes / diabetic kidney disease to WM physician (around 30 mins)
    i. Have you encountered any patients enquiring about the use of WM for diabetes or related complications?
        - Frequency (many?)
        - What do they ask? (Referral? Efficacy? Safety?)
        - Patient's stage of disease (with / without complications, CKD stage?)
    ii. How would you feel about referral / use of WM and answer the patients in daily practice?
    iii. Have you encountered any patients with undesired medical behaviour (eg. Reluctant to comply with CM) / suspected WM induced complications?
        - How do they present?
        - How would you determine the relationship?

3. Concern on referral (around 30 mins)
    i. How do you perceive the effect and toxicity of WM / CM for diabetes and diabetic kidney disease in general?
    ii. What would you consider in advising patient referral / management for diabetes?
        - Any obstacles in consideration? Any further information / assistance / tools needed?
    iii. What do you expect the role / attitude of WM physician?
    iv. Any suggested research area for CM / WM?
        - How do you perceive DKD as a disease?
        - Common herbs?
        - Herbs that are likely to be / reported to be nephrotoxic?
        - Clinical protocol?
        - Responder analysis?
    v. Any suggested evidence dissemination form / platform?
        - Clinical practice guideline?
        - Web?
        - CME course / seminar?

4. Summary, conclusion and acknowledgement (5 mins)

Supplementary File

For patient:

1. **Understanding of own health condition (ice-breaking, 5 mins)**
   i. Do you think you understand your own health condition?
      - What to follow up at the clinic?

2. **Experience on referral of diabetes / diabetic kidney disease (30 mins)**
   i. Have you ever used / interested in using CM as treatment of diabetes / DKD?
   ii. If yes:
      - What attracts you?
         - Dissatisfaction over conventional method / service / current health status?
         - Autonomy over own health?
         - Belief in IM?
      - Where did you have the CM consultation?
      - ****What do you expect from the CM physician?**
         - **Concrete examples, eg. delay dialysis / transplant, better glycemic control, symptomatic relief (eg fatigue), cure, less WM medication?**
      - How do you choose the CM physician?
         - Experience? Academic qualification? knowledge on WM? affordability/accessibility? Service quality?
      - How do you feel after the treatment?
         - Condition improved? (subjective feeling? Lab test?)
         - Able to complement each other?
         - Is the service satisfactory? (Quality, accessibility?)
         - Any room for improvement?
         - Any adverse feeling?
      - **Did WM physician asked about CM use?
      - ****Did you inform the WM physician? Why? (Attitude? Knowledge? Restriction?)**
      - Did any CM / WM physician advise against WM / CM?
   iii. If not → Proceed to 3) concern on referral
   iv. Would you add CM to your daily diet? (北芪, 黨參, 蓮子, 百合...etc)

3. **Concern on referral (around 30 mins)**
   i. Have you ever consulted WM / CM physician about CM efficacy
   ii. What would you consider before enquiring / requesting advice on referral?
      - Interest on referral?
      - Clinical need?
      - Attitude / feedback / action from WM/CM physician?
   iii. What advice would you expect from the physician?
   iv. What information would you like to know further?
   v. How would you get medical information? (From doctor? TV? Radio? Newspaper?)
   vi. How would do you feel about the attitude of WM/CM physician on referral?
   vii. Do you think WM / CM physician know each other / should know each other more?
